# Shear Strain Singularity-Inspired Identification of Initial Delamination in CFRP Laminates: Multiscale Modulation Filter for Extraction of Damage Features

**DOI:** 10.3390/polym14112305

**Published:** 2022-06-06

**Authors:** Wei Xu, Yunfeng Lu, Ruihu Zhu, Maciej Radzieński, Maosen Cao, Wiesław Ostachowicz

**Affiliations:** 1 Department of Engineering Mechanics, Hohai University, Nanjing 210098, China; yflu@hhu.edu.cn; 2 Jiangsu Province Wind Power Structural Engineering Research Center, Hohai University, Nanjing 210098, China; 3 College of Harbour, Coastal and Offshore Engineering, Hohai University, Nanjing 210098, China; ruihuzhu@hhu.edu.cn; 4 Institute of Fluid-Flow Machinery, Polish Academy of Sciences, 80-231 Gdańsk, Poland; maciej.radzienski@imp.gda.pl (M.R.); wieslaw.ostachowicz@imp.gda.pl (W.O.); 5 College of Civil and Architecture Engineering, Chuzhou University, Chuzhou 239000, China

**Keywords:** CFRP laminate, identification of initial delamination, shear strain singularity, multiscale modulation filter, non-contact laser measurement

## Abstract

Identification of initial delamination is crucial to ensure the safety of the fiber-reinforced laminated composite structures. Amongst the identification approaches based on mode shapes, the concept of multiscale shear-strain gradient (MSG) has an explicit physical sense of characterizing delamination-induced singularity of shear strains; moreover, it is robust against noise interference owing to the merits of multiscale analysis. However, the capacity of the MSG for identifying initial delamination is insufficient because the delamination-induced singularity peak can be largely obscured by the global component of the MSG. Addressing this problem, this study proposes an enhanced approach for identifying initial delamination in fiber-reinforced composite laminates. In particular, the multiscale modulation filter (MMF) is proposed to modulate the MSG with the aim of focusing on damage features, by which a new concept of enhanced MSG (EMSG) is formulated to extract damage features. By taking advantage of the MMF with the optimal frequency translation parameters, the EMSG is concentrated in a narrow wavenumber band, which is dominated by the damage-induced singularity peak. As a consequence, the delamination-induced singularity peak in the EMSG can be isolated from the global component. The capacity of the approach for identifying initial delamination is experimentally validated on a carbon fiber reinforced polymer (CFRP) laminate, whose mode shapes are acquired via non-contact laser measurement. The experimental results reveal that the EMSG-based approach is capable of graphically characterizing the presence, location, and size of initial delamination in CFRP laminates.

## 1. Introduction

Carbon fiber reinforced polymer (CFRP) laminates have been increasingly utilized in structural components such as wind turbine blades owing to their appealing advantages of high strength, low density, and anti-corrosion. However, transverse impacts can cause local delamination (debonding of adjacent plies) in CFRP laminates. As a type of barely visible impact damage (BVID), delamination can hardly be judged from the appearance of the fiber-reinforced composite laminates [1]. To prevent delamination from developing to severe degrees, it is of significance to identify delamination at the early stage.

Utilizing local changes in physical properties caused by delamination, nondestructive testing (NDT) techniques have been widely applied for the detection of delamination in fiber-reinforced composite laminates [2,3,4,5,6,7,8,9,10,11,12,13,14,15,16,17]. Recently, ultrasonic waves have been utilized for the identification of delamination by capturing characteristics of waves [18,19,20,21,22,23,24,25,26,27]. More recently, with the aid of the laser scanning technique, mode shapes of fiber-reinforced composite laminates can be acquired by measuring full-field operating deflection shapes (ODSs) at resonances. Existing researches suggest that delamination can induce discontinuities in derivatives of mode shapes with respect to spatial coordinates, and such discontinuities can become ideal indicators to characterize the presence, location, and size of the delamination. Representative studies are as follows: Inspired by the delamination-induced singularity in normal strains, curvature mode shapes (CMSs) have been widely utilized for the identification of delamination in fiber-reinforced composite laminates. Three typical algorithms based on mode shapes are compared by Qiao et al. [28] to evaluate their capacities for identifying delamination in CFRP laminates. Araújo dos Santos et al. [29] used acoustics to excite a CFRP laminate and measured its mode shapes via TV holography. The difference between CMSs under structurally intact and damaged statuses was utilized for the identification of delamination caused by the impact. Pérez et al. [30] proposed a curvature damage factor based on the integrated utilization of natural frequencies and CMSs, by which the delamination in CFRP laminates can be pinpointed. The complex-wavelet CMS was formulated by Xu et al. [31] for the identification of delamination in CFRP laminates. Superior to the conventional CMS, sensitivity to delamination and robustness against noise interference is enhanced in the complex-wavelet CMS. The Fourier spectral CMS was formulated by Yang et al. [32,33] through integrating the Fourier spectral method into the conventional CMS, whose capacity for identifying delamination was verified in the CFRP laminates. By means of continuous laser scanning, Chen et al. [34] and Xu et al. [35] used local anomalies in mode shapes for the identification of delamination in CFRP laminates. Inspired by the delamination-induced singularity of shear strains, Cao et al. [36] formulated the concept of a multiscale shear-strain gradient (MSG) for identifying delamination in glass-fiber-reinforced polymer (GFRP) laminates. Nevertheless, it is difficult to identify initial delamination using the MSG, because the stiffness reduction in the delamination region is slight [37]. As a consequence, slight singularity peaks caused by initial delamination can be obscured by the global components of MSGs and become ambiguous for the identification of initial delamination. With this concern, the Teager–Kaiser modal energy [38,39] was formulated to evaluate the point-wise energy of a mode shape. Local changes in the Teager–Kaiser modal energy can be utilized to identify initial delamination. Furthermore, the approach’s robustness against noise interference is enhanced by integrating multi-resolution analysis into the Teager–Kaiser modal energy. Recently, a damage index was built on the equation of out-of-plane motion, representing the delamination-induced perturbation to the dynamic equilibrium [40]. The applicability of the damage index was validated on a CFRP laminate with initial delamination.

The MSG-based approach is enhanced in this study for identifying initial delamination in fiber-reinforced composite laminates. In particular, a multiscale modulation filter (MMF) is proposed to modulate the MSG with the aim of focusing on damage features. On the basis of the MMF, a new concept of enhanced MSG (EMSG) is formulated to extract damage features. By taking advantage of the MMF with the optimal frequency translation parameters, the EMSG is concentrated in a narrow wavenumber band, which is dominated by the damage-induced singularity peak.

The rest of the paper is organized as follows: Section 2 proposes an EMSG-based approach for the identification of initial delamination in fiber-reinforced composite laminates. Section 3 experimentally validates the capacity of the approach on a CFRP laminate that bears initial delamination. A scanning laser vibrometer (SLV) is utilized to measure its mode shapes via non-contact laser measurement. Section 4 presents the conclusions.

## 2. EMSG-Based Approach for Identification of Initial Delamination in Fiber-Reinforced Composite Laminates

### 2.1. Identification of Delamination by Singularity of Shear Strain

For a Kirchhoff plate with in-plane coordinates x and y and transverse coordinate z, the distribution of engineering shear strains $\gamma_{xy}$ for an out-of-plane mode shape W(x,y) can be written as [41]:

(1) $$\gamma_{xy}=-2z\frac{\partial^{2}W(x,y)}{\partial x\partial y}.$$

Local damage such as delamination can cause singularity of shear strains, i.e., local discontinuity of the derivatives of shear strains. Therefore, the concept of SG was proposed to characterize the delamination-induced singularity of shear strains, denoted as ${\overset{↔}{\gamma }}_{xy}$ [36]:

(2) $${\overset{↔}{\gamma }}_{xy}=-2z\frac{\partial^{4}W(x,y)}{\partial x^{2}\partial y^{2}}.$$

Inspired by the physical sense of the SG, a damage index (DI), denoted as DI(x,y), was established to characterize the delamination-induced singularity of surficial shear strains in a fiber-reinforced composite laminate:

(3) $$DI(x,y)=\frac{\partial^{4}W(x,y)}{\partial x^{2}\partial y^{2}},$$

by which the presence, location, and size of the delamination can be characterized in a graphical manner. 

### 2.2. Multiscale Analysis for Elimination of Noise Components

Although the SG has an explicit physical sense for the identification of delamination in fiber-reinforced composite laminates, direct use of the DI by Equation (3) could lead to intensive noise interference because noise components in densely measured mode shapes can be amplified significantly owing to differential operation [42].

Addressing this problem, integration of the SG and multiscale analysis produces the concept of MSG, denoted as ${\overset{↔}{\gamma }}_{xy}^{s}$:

(4) $${\overset{↔}{\gamma }}_{xy}^{s}={\overset{↔}{\gamma }}_{xy}⊗{\bar{g_{2,2}}}_{s},$$

based on which the multiscale DI (MDI), denoted as $MDI_{s}(u,v)$, was established by wavelet transform [43]:

(5) $$MDI_{s}(u,v)=W⊗{\bar{g_{2,2}}}_{s}(u,v)=s^{4}\frac{\partial^{4}}{\partial u^{2}\partial v^{2}}(W⊗{\bar{g}}_{s})(u,v),$$

where $g(x,y)=\frac{1}{2\pi \sigma_{x}\sigma_{y}}e^{-(x^{2}/2\sigma_{x}^{2}+y^{2}/2\sigma_{y}^{2})}$ is the Gaussian function with $\sigma_{x}$ and $\sigma_{y}$ denoting the standard variances in the *x*- and *y*-directions, respectively, $g_{2,2}(x,y)=\frac{\partial^{4}g(x,y)}{\partial x^{2}\partial y^{2}}$, ${\bar{g_{2,2}}}_{s}(x,y)=\frac{1}{s}g_{2,2}(\frac{-x}{s},\frac{-y}{s})$, s is the scale parameter, and u and v are the spatial translation parameters in the *x*- and *y*-directions. The essence of the relationship between Equations (3) and (5) is that W in Equation (3) is convolved with ${\bar{g}}_{s}(x,y)=\frac{1}{s}g(\frac{-x}{s},\frac{-y}{s})$ and scaled by $s^{4}$ in Equation (5). The principle of multiscale analysis for an MSG is as follows [43]: the high-frequency noise components and low-frequency global component of the MSG are associated with small and large scales, respectively. By gradually increasing the scale parameter, the noise components at small scales can be averaged in ever-wider scaled Gaussian windows and gradually eliminated. Meanwhile, delamination-induced singularity can be retained with the increase in the scale parameter. When the scale parameter reaches its optimal value, which is between the scales associated with noise and global components, only the delamination-induced singularity peak arises in the MSG. In consequence, the singularity peak can manifest the presence of the delamination. Moreover, the spatial distribution of the singularity peak can characterize the location and size of the delamination. 

### 2.3. MMF for Extraction of Initial Delamination Features

Although the delamination-induced singularity of shear strains is retained in the MSG, the wavenumber band associated with the singularity peak is narrow. As a result, it is difficult to find the optimal scale parameter to thoroughly isolate the delamination-induced singularity peak from the global component of the MSG, leading to the mixed distribution of the aforementioned components in the MSG. Unfortunately, in most situations, singularity peaks induced by initial delamination can be largely obscured by the global components of MSGs. Therefore, the capacity of the MSG for identifying initial delamination in fiber-reinforced composite laminates is insufficient. 

To overcome this barrier, the MMF is proposed in this study to enhance the MSG with the aim of focusing on and extracting damage features by isolating singularity peaks from global components: the Gabor function G(x,y) is utilized to replace the Gaussian function g(x,y) in Equation (5), which leads to a new concept of EMSG. On the basis of the EMSG, an enhanced MDI (EMDI) is established for the identification of initial delamination in fiber-reinforced composite laminates, denoted as $EMDI_{s}(u,v)$:

(6) $$EMDI_{s}(u,v)=W⊗{\bar{G_{2,2}}}_{s}(u,v)=s^{4}\frac{\partial^{4}}{\partial u^{2}\partial v^{2}}(W⊗{\bar{G}}_{s})(u,v),$$

where $G(x,y)=g(x,y)e^{i(\gamma x+\eta y)}$ is the Gabor function with i denoting the imaginary unit and γ and η denoting the frequency translation parameters in the *x*- and *y*-directions, respectively, $G_{2,2}(x,y)=\frac{\partial^{4}G(x,y)}{\partial x^{2}\partial y^{2}}$, ${\bar{G_{2,2}}}_{s}(x,y)=\frac{1}{s}G_{2,2}{}^{*}(\frac{-x}{s},\frac{-y}{s})$ with $G_{2,2}{}^{*}$ denoting the complex conjugate of the mother wavelet $G_{2,2}(x,y)$. The essence of the relationship between Equations (5) and (6) is that ${\bar{g}}_{s}(x,y)$ is multiplied by the MMF $e^{i(\gamma \frac{-x}{s}+\eta \frac{-y}{s})}$ to produce ${\bar{G}}_{s}(x,y)=\frac{1}{s}G(\frac{-x}{s},\frac{-y}{s})$. Thereby, the MSG can be modulated by the MMF to concentrate in the narrow wavenumber band, which is dominated by the delamination-induced singularity peak. By taking advantage of the MMF with the optimal frequency translation parameters γ and η, the EMSG is capable of focusing on the singularity peak, such that the features of initial delamination can be extracted. As a consequence, the delamination-induced singularity peak can be readily isolated from the global component of the MSG.

The 2D Gabor function G(x,y) can be written as the product of two 1D Gabor functions in the *x*- and *y*-directions:

(7) $$G(x,y)=G(x)G(y)=g(x)e^{i\gamma x}g(y)e^{i\eta y}.$$

The 1D Gabor function G(x) is formulated by multiplying the 1D Gaussian function g(x) by a complex exponential function $e^{i\gamma x}$:

(8) $$G(x)=g(x)e^{i\gamma x}.$$

As per Equation (7), $G_{2,2}(x,y)$ can be expressed as:

(9) $$\begin{array}{l} G_{2,2}(x,y)=\frac{\partial^{4}G(x,y)}{\partial x^{2}\partial y^{2}}=\frac{\partial^{4}[G(x)G(y)]}{\partial x^{2}\partial y^{2}}=\frac{\partial^{4}[g(x)e^{i\gamma x}g(y)e^{i\eta y}]}{\partial x^{2}\partial y^{2}}\\ \;=(\frac{d^{2}g(x)}{dx^{2}}-\gamma^{2}g(x)+2\gamma \frac{dg(x)}{dx}i)(\frac{d^{2}g(y)}{dy^{2}}-\eta^{2}g(y)+2\eta \frac{dg(y)}{dy}i)e^{i(\gamma x+\eta y)}. \end{array}$$

As a mother wavelet, $G_{2,2}(x,y)$ satisfies the zero-mean condition [43]:

(10) $$\int_{-\infty }^{\infty }\int_{-\infty }^{\infty }G_{2,2}(x,y)dxdy=0.$$

It is noteworthy that the damage features contained in an EMDI heavily depend on the mode shape one selects. In that situation, a single mode is insufficient to characterize full details of the damage. With this concern, an integrating scheme is utilized in this study for the identification of initial delamination in fiber-reinforced composite laminates, by which the fused EMDI (FEMDI) is established with the intersection of multiple EMDIs [44], denoted as $FEMDI_{s}(x,y)$:

(11) $$FEMDI_{s}(x,y)=\overset{N}{\underset{n=1}{\cap }}EMDI_{s}^{n}(x,y).$$

## 3. Proof of Concept

The capacity of the EMSG-based approach for identifying initial delamination is experimentally validated via non-contact laser scanning of vibration using an SLV. It is noteworthy that although the experimental specimen in this study is a CFRP laminate, the applicability of the approach can be extended to other fiber-reinforced composite laminates, including GFRP laminates.

### 3.1. Experimental Setup and Specimen

**Figure 1:**
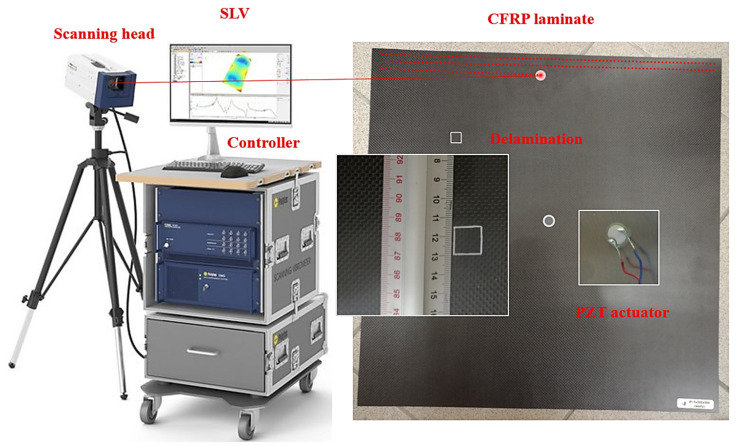
Experimental setup and specimen.

Take an eight-ply CFRP symmetric cross-ply laminate as an experimental specimen, whose fiber orientations are 0/90°. The plane dimensions of the laminate are 500 × 500 mm^2^ in the *x*- and *y*-directions, respectively; the thickness of the laminate is 3 mm in the *z*-directions. For the sake of description, the surface belonging to the first ply is defined as the front surface of the specimen, and the reverse side is defined as the back surface. To manufacture initial delamination, a square Teflon sheet was inserted between the interfaces of the second and third plies when the specimen was fabricated. The delamination is almost invisible, even from the zoomed-in view of the specimen surface. On the front surface of the specimen, white outlines are marked to indicate the delamination region. The dimensions of the initial delamination are 15 × 15 mm^2^. The delamination spans from 117.5 to 132.5 mm in the *x*-direction and 367.5 to 382.5 mm in the *y*-direction, the center of which is located at *x* = 125 mm and *y* = 375 mm. In the dimensionless coordinates ζ=x/500 and η=y/500, the center of the delamination is located at ζ=0.25 and η=0.75, and the delamination area spans from 0.235 to 0.265 in ζ and 0.735 to 0.765 in η. Figure 1 shows the experimental setup and specimen. Suspended by two strings in the upper corners, the boundary conditions of the specimen at four edges are considered free. Note that the approach is applicable to arbitrary boundary conditions of fiber-reinforced composite laminates. On the front surface of the specimen, a circular lead zirconate titanate (PZT) actuator (10 mm in diameter) is mounted in its geometrical center to generate high-frequency harmonic waves to excite the CFRP laminate. Simultaneously, an SLV (PSV-400, Polytec, Waldbronn, Germany) scans the entire back surface (covered by reflection tapes) in rows from top to bottom. The measurement grid on the back surface is 375 × 375 with a uniform interval of 1.33 mm in both *x*- and *y*-directions.

### 3.2. Experimental Results

**Figure 2:**
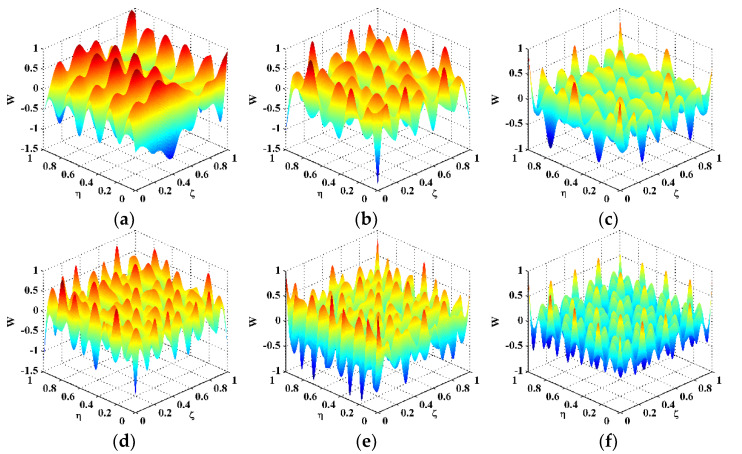
Mode shapes of the CFRP laminate for Scenarios (**a**) I, (**b**) II, (**c**) III, (**d**) IV, (**e**) V, and (**f**) VI.

**Figure 3:**
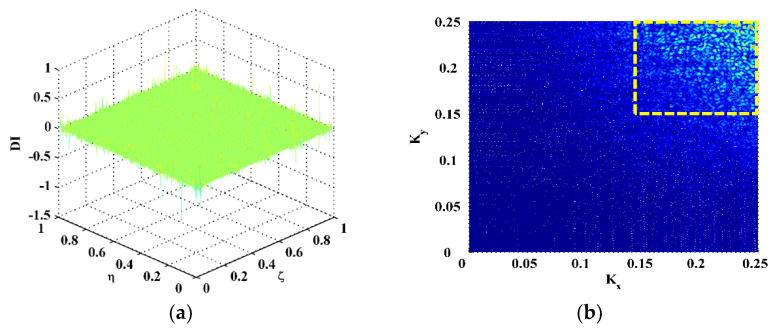
(**a**) DI and its (**b**) wavenumber spectrum for Scenario V.

**Figure 4:**
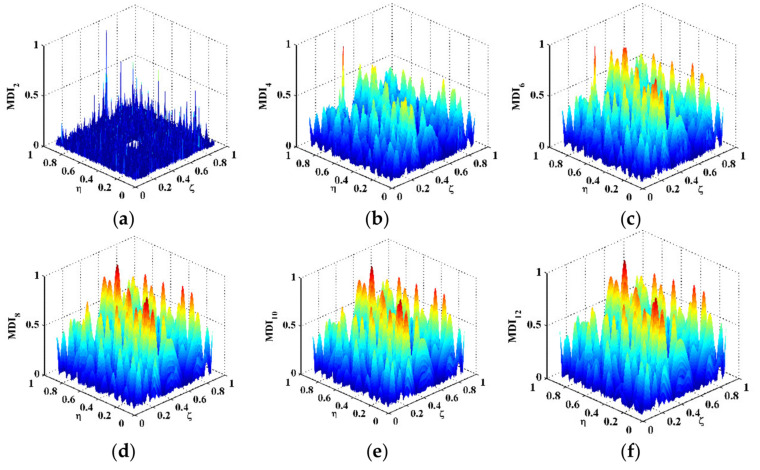
MDIs for Scenario V with scale parameters of (**a**) 2, (**b**) 4, (**c**) 6, (**d**) 8, (**e**) 10, and (**f**) 12.

**Figure 5:**
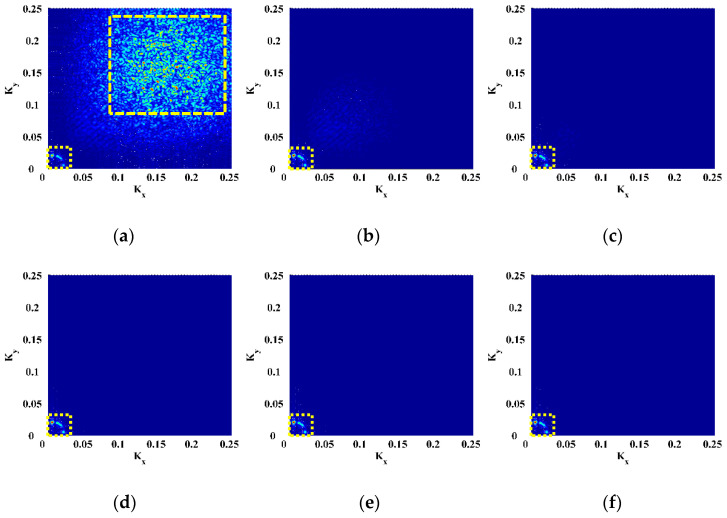
Wavenumber spectra of the MDIs for Scenario V with scale parameters of (**a**) 2, (**b**) 4, (**c**) 6, (**d**) 8, (**e**) 10, and (**f**) 12.

Six natural frequencies, i.e., 1260.94, 1639.06, 2346.88, 3184.38, 4142.19, and 4440.63 Hz, are arbitrarily selected after modal analysis. The corresponding mode shapes are utilized for experimental validation, denoted as Scenarios I–VI. The CFRP laminate is excited at these frequencies to pump more energy into it, such that a higher signal-to-noise ratio can be achieved owing to the higher magnitudes of vibration. The ODSs associated with the natural frequencies approximate the corresponding mode shapes of this lightly damped specimen. Therefore, the measured ODSs are regarded as the mode shapes, which are shown in Figure 2. Note that the ODSs are normalized, with their maximum amplitudes being units. Taking Scenario V for illustration, the DI is obtained by Equation (3) using the finite difference method and shown in Figure 3a. It can be seen from Figure 3a that noise components dominate the DI, whereas the delamination-induced singularity peak is masked by noise interference and can barely be identified. To investigate components of the DIs with different wavelengths, the 2D Fourier transform is adopted to obtain their wavenumber spectra:

(12) $$\hat{f}(k_{x},k_{y})=\int_{-\infty }^{\infty }\int_{-\infty }^{\infty }f(x,y)e^{-i2\pi (k_{x}x+k_{x}y)}dxdy,$$

in which a signal f(x,y) in the spatial domain is transformed to $\hat{f}(k_{x},k_{y})$ in the wavenumber domain with $k_{x}$ and $k_{y}$ denoting the wavenumbers in the *x*- and *y*-directions (higher wavenumbers indicate lower wavelengths). By 2D Fourier transform, the DI for Scenario V is mapped to the wavenumber domain and shown in Figure 3b, in which only noise components appear at high wavenumbers (highlighted by a dashed rectangle). Without losing generality, one can divide the wavenumbers $k_{x}$ and $k_{y}$ by $K_{x}$ and $K_{y}$, respectively, which are the spatial sampling frequencies in the *x*- and *y*-directions. By Equation (5), the DI is transformed to MDIs with an increase in scale parameters from 2 to 12 (Figure 4), the wavenumber spectra of which are obtained by Equation (12) and shown in Figure 5. To remove distortions produced by the wavelet transform [36], values of the MDIs vanish in the vicinity of the boundaries and geometrical center where the PZT actuator is mounted. As can be seen from $MDI_{2}$ with s=2 (Figure 4a), noise interference is less pronounced, and a global component appears; in its wavenumber spectrum in Figure 5a, besides the noise components highlighted by a dashed rectangle, the global component highlighted by a dotted rectangle appears at low wavenumbers. In $MDI_{4}$ with s=4 (Figure 4b), noise components are further suppressed; meanwhile, the delamination-induced singularity peak arises. However, the singularity peak is surrounded by the global component and becomes ambiguous to indicate the delamination. In the wavenumber spectrum of $MDI_{4}$ (Figure 5b), the features of the singularity peak are shadowed by the global component. By further increasing the scale from 6 to 12, the singularity peaks are totally obscured by the global components (Figure 4c–f), and only the global components can be identified in the wavenumber spectra of the MDIs (Figure 5c–f). In consequence, it is difficult to search for the optimal scale parameter at which the delamination-induced singularity peak can be isolated from the global component.

**Figure 6:**
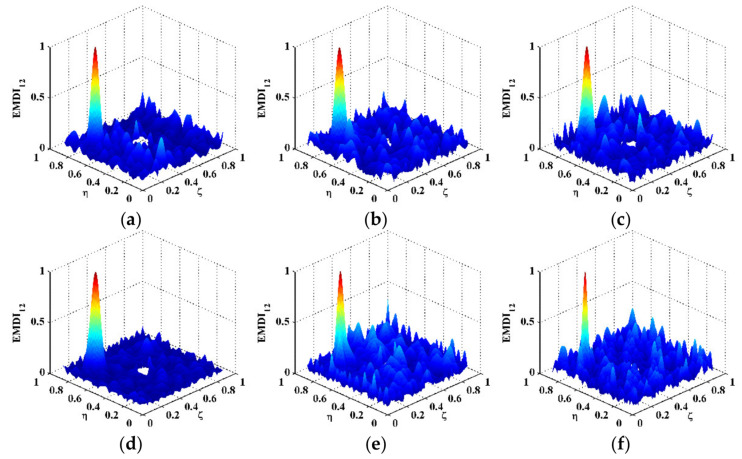
EMDIs for Scenarios (**a**) I, (**b**) II, (**c**) III, (**d**) IV, (**e**) V, and (f) VI.

**Figure 7:**
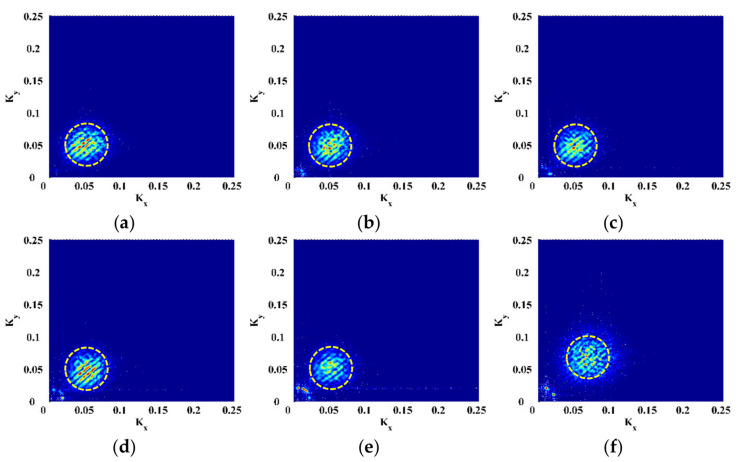
Wavenumber spectra of EMDIs for Scenarios (**a**) I, (**b**) II, (**c**) III, (**d**) IV, (**e**) V, and (**f**) VI.

**Figure 8:**
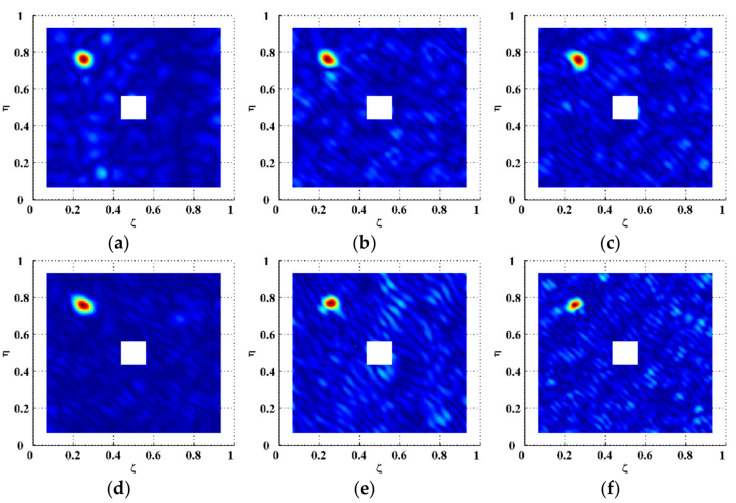
Planforms of the EMDIs for Scenarios (**a**) I, (**b**) II, (**c**) III, (**d**) IV, (**e**) V, and (**f**) VI (values vanish in the vicinity of boundaries and geometrical center).

**Figure 9:**
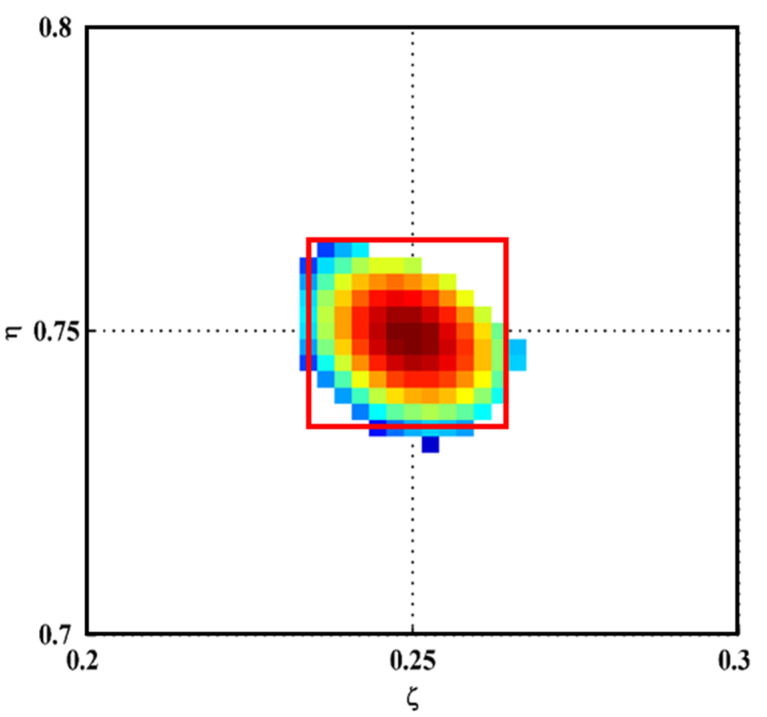
Zoomed-in planform of the FEMDI (outlines of the actual region of the delamination are marked in red).

To isolate delamination-induced singularity peaks, the MDIs are enhanced by Equation (6) to produce the EMDIs, as shown in Figure 6. The optimal frequency translation parameters γ and η are determined to be three after trials. It can be seen that in each EMDI, noise and global components are basically eliminated. Meanwhile, the delamination-induced singularity peak is retained and concentrated in the delamination region. In the wavenumber spectra of the EMDIs (Figure 7), it can be found that features highlighted by dashed circles evidently indicate the presence of the delamination-induced singularity peaks, the wavenumber bands of which are between those of noise and global components, as shown in Figure 6. As can be seen from Figure 8, the singularity peaks in the planforms of the EMDIs clearly pinpoint the initial delamination: the identified delamination is centered at ζ=0.25 and η=0.75. To characterize full details of the delamination, the EMDIs are fused by Equation (11) to obtain the FEMDI, whose zoomed-in planform is shown in Figure 9. The FEMDI clearly indicates that the identified delamination spans from about 0.235 to 0.265 in ζ, and about 0.735 to 0.765 in η, which corresponds well with the actual region of the delamination, whose outlines are marked in red. Hereby, superior to the MSG-based approach, the EMSG-based approach is capable of graphically characterizing the presence, location, and size of the initial delamination. 

## 4. Conclusions

Inspired by the delamination-induced singularity of shear strains, MSGs have been utilized for the identification of delamination in fiber-reinforced composite laminates; however, insensitivity to initial delamination hinders its applicability to the early-stage diagnosis of laminated composite structures. To overcome this barrier, the MMF is proposed to modulate the MSG, by which a new concept of EMSG is formulated for the identification of initial delamination in fiber-reinforced composite laminates. The capacity of the approach is experimentally validated on a CFRP laminate with initial delamination, whose mode shapes are acquired via non-contact laser measurement using an SLV. The experimental results reveal that the EMSG-based approach can graphically characterize the presence, location, and size of the initial delamination. Some conclusions are as follows.

(1) Due to the difficulty in finding the optimal scale parameter of the MSG, the singularity peak induced by initial delamination cannot be thoroughly isolated from the global component. In consequence, the singularity peak can be largely obscured by the global component, which hinders the capacity of the MSG for identifying initial delamination in fiber-reinforced composite laminates.
(2) The MMF with the optimal frequency translation parameters can enhance the EMSG to concentrate in a narrow wavenumber band, which is dominated by the damage-induced peak. In that situation, the EMSG can focus on the damage features and extract them for the identification of initial delamination. To be specific, the delamination-induced peak in the EMSG can be isolated from the global component, whereby the presence, location, and size of the initial delamination can be graphically characterized.
(3) The EMDI built on a single mode shape may contain insufficient features of the initial delamination. To extract the full features of the initial delamination, an integrating scheme is utilized to fuse the delamination-induced singularity peaks associated with multiple mode shapes, whereby the FEMDI is formulated with the intersection of multiple EMDIs.

## Data Availability

Not applicable.
